# The Economic burden of opioid-related serious adverse events and undertreatment of acute pain in the United States

**DOI:** 10.3389/fpubh.2026.1824038

**Published:** 2026-06-17

**Authors:** James C. Hackworth, John E. Schneider, Maggie L. Do Valle, David S. Fam, Robert Ohsfeldt, Emanuela Offidani, Jim Potenziano

**Affiliations:** 1 Tris Pharma, Monmouth Junction, NJ, United States; 2 Avalon Health Economics, Morristown, NJ, United States; 3 Avalon Health Economics, Miami, FL, United States; 4 Texas A&M University, College Station, TX, United States; 5 Clinical Epidemiology and Evaluative Sciences, Weill Cornell Medicine, New York, NY, United States

**Keywords:** acute pain, chronification of pain, economic burden, healthcare utilization, opioid use disorder (OUD), pain management, undertreatment

## Abstract

Acute pain has become increasingly prevalent in the United States due to an aging population, rising metabolic disease rates, and growth in surgical procedures. Opioids remain the primary treatment for moderate-to-severe acute pain but are associated with serious adverse events, including misuse, dependency, opioid use disorder, and overdose. In contrast, restrictive prescribing may result in undertreatment of acute pain and progression to chronic pain (“chronification”), generating additional long-term costs. As an accompanying analysis to our prior work on the increasing burden of acute pain in the wake of the opioid crisis, this study estimates the annual U.S. economic burden attributable to opioid-related serious adverse events and undertreatment of acute pain, including chronification. An economic analysis was performed to estimate direct healthcare costs and indirect productivity losses, with all values adjusted to 2024 U.S. dollars. The total annual economic burden was estimated at $425.0 billion (range: $164.5–$1,215.5 billion). Direct costs were driven by dependency, misuse/abuse, OUD, and chronic pain, while productivity losses were dominated by fatal overdoses and chronification. These findings highlight the substantial economic consequences of both opioid-related harms and inadequate pain management, demonstrating the urgent need for pain management strategies that adequately treat acute pain while minimizing the risk of serious adverse events.

## 1 Introduction

The burden of acute pain in the United States has increased substantially over the past two decades (1). As a result, effective pain management remains a significant clinical and public health priority, yet clinicians and patients face a fundamental dilemma. On one hand, opioids continue to play a necessary role in the treatment of moderate to severe acute pain despite their associated risks and adverse events. On the other hand, efforts to reduce opioid-related harms risk contributing to the undertreatment of acute pain with subsequent long-term consequences, including transition of acute pain to chronic pain (typically referred to as the “chronification” of pain). This tension underscores the need for a more nuanced understanding of how pain management practices influence health outcomes and contribute to the economic burdens of pain and the adverse effects of opioid use.

Accurately estimating the economic burden of pain management remains challenging, as costs vary considerably depending on the type, intensity, and the success of treatment. Nevertheless, estimates are substantial and include healthcare expenditures, productivity losses, and broader societal costs (2).^1^ Extant analyses focus on the cost of treating and consequences of having pain, but not secondary costs associated with existing treatment options. Notably, these evaluations have largely discounted the costs of chronification and the economic consequences associated with the undertreatment of acute pain (3–5).

The objective of this analysis is to address these gaps in the literature by estimating the healthcare and productivity costs associated with limited treatment options for moderate to severe pain, including costs related to opioid dependency, addiction, serious adverse events (SAEs), undertreatment of acute pain, and pain chronification (1).

By quantifying the burden of both scenarios associated with opioid prescribing behaviors, this analysis identifies the key economic drivers more precisely to inform the development of targeted strategies to address both the risks of opioid-related health problems and the consequences of undertreated pain due to inadequate treatment, ultimately highlighting the need for new therapeutic alternatives for pain management.

## 2 Background

While the exact prevalence of acute pain in the general population remains difficult to quantify, a study using data from the National Health and Nutrition Examination Survey estimated that about 12% of Americans were experiencing acute pain at the time of the survey (6). Over the past two decades, acute pain has become increasingly common, due primarily to more surgeries, an aging population, and the rapid growth in the prevalence of metabolic disease. For example, individuals with obesity and metabolic disorders face a substantially higher likelihood of experiencing acute and chronic pain, with obese patients more than twice as likely to report severe acute pain compared to normal weight individuals (7) and bearing higher surgical burden, with reports showing that up to 72% of patients undergoing total knee or total hip replacement were obese, and obese patients were nearly twice as likely to require revision surgery due to significantly higher postoperative complications, including deep infection (8, 9). Furthermore, despite the recent market introduction of GLP-1 receptor agonists, population-level obesity rates decreased minimally, from 39 to 37% (10). This modest decrease indicates that obesity-related complications will continue to challenge the delivery of effective acute pain management for the long term.

Although opioids are often the only effective treatment for moderate to severe acute pain, even brief use comes with substantial risks such as physical dependence, euphoria, risk of overdose, and respiratory depression, which together can lead to significant adverse outcomes. Critically, a meaningful proportion of the patients currently suffering from opioid use disorder and those dying from opioid overdoses had their first exposure within the healthcare system seeking treatment for acute or chronic pain (11). Beyond individual outcomes, opioids are associated with substantial humanistic and economic burden, largely driven by the risk of misuse and diversion, both of which have the potential to exacerbate the risks associated with dependence and overdose (1). Conversely, refraining from using opioids in cases where other treatment options prove inadequate, creates a different set of risks including the risk of chronification, with prior studies suggesting that approximately 20% of patients experience this progression (12–14).

Clinical and regulatory efforts have achieved incremental progress lowering addiction risks while unintendedly increasing the burden of untreated pain. The historical evolution of pain management clearly illustrates the polarization of clinical practice. First, the rapid increase in rates of opioid prescribing following the American Pain Society’s “Pain is the Fifth Vital Sign” campaign in 1996 contributed to rising rates of opioid dependency and overdose (15). This prompted the CDC to introduce opioid prescribing guidelines in 2016 to promote more cautious prescribing for acute and chronic pain with the intent of reducing opioid-related health problems. By 2024, per capita prescription opioid use had declined by 69% from its peak in 2011 (16). This dramatic shift contributed to more restrictive prescribing behaviors among healthcare professionals, reduced flexibility in dosage and duration, and increased reluctance to initiate opioid therapy even when clinically appropriate (1, 17–20). Additionally, state-level prescribing regulations often mandated specific prescribing limits, many of which were informed by CDC recommendations. These measures have reduced opioid prescribing overall, but their rigid approaches limiting both inappropriate and appropriate opioid therapy led to unintended consequences, including undertreatment of acute pain (21).

Second, in recent years, several non-opioid therapeutic approaches have been approved or advanced in late-stage development, reflecting efforts to expand treatment options beyond opioids. One recent example is the FDA approval of suzetrigine (Journavx®), a novel oral non-opioid analgesic targeting peripheral sodium channels (22). Based on data from pivotal trials in acute pain (23), suzetrigine may be able to replace some opioid use, either alone or potentially in combination with other analgesics as part of multi-modal therapy. However, it is likely that many acute pain patients will still require opioids, particularly those with more severe pain.

Third, a recent policy, the NOPAIN Act, went into effect on January 1, 2025. It requires Medicare to make separate payments for qualifying non-opioid drugs and devices in outpatient surgical settings (48). The unbundling of treatment of surgical payments creates incentives for hospitals, through reimbursement, to use non-opioid modalities, as some were more expensive upfront. However, this policy will only be effective if there is availability of non-opioid therapeutics that can treat pain as effectively as opioids. Unfortunately, although the FDA has issued draft guidance to accelerate the development of non-opioid treatments for pain, acknowledging ongoing reliance on opioids amid limited alternatives (24) with few exceptions, such non-opioid therapeutics do not yet exist. Thus, this policy also risks inadvertently exacerbating the undertreatment of pain observed in multimodal analgesia (MMA). While MMA was intended to reduce opioid consumption through the use of ‘opioid-sparing’ agents, the lack of potent non-opioid options often resulted in reduced efficacy (49, 50). By prioritizing the avoidance of opioids over the attainment of adequate pain control, these protocols frequently leave patients with sub-therapeutic relief, transforming a safety strategy into a driver of clinical undertreatment (25). Therefore, there is an urgent need for safer and less addictive therapeutics that can treat more severe pain.

## 3 Limitations in current economic estimates

The attributable costs of pain, including pain treatment, have been well-studied and found to vary considerably depending on causal pathways and type and intensity of treatment. Gaskin and Richard provided some broad estimates using data from the Medical Expenditure Panel Survey (MEPS), finding that the attributable “excess” healthcare costs of pain (acute and chronic) summed to nearly $300 billion in 2010 dollars, or $436 billion in 2024 dollars (2). Costs related to work productivity, as measured by absenteeism, presenteeism, and depressed wages, added another $487 billion, bringing the total attributable cost of pain to $923 billion in 2024 dollars. Their analysis also found that moderate pain doubled healthcare costs, whereas severe pain tripled these costs. Moreover, private insurers continue to bear the largest shares of healthcare costs attributable to pain, accounting for approximately 43% of the total expenditure (26). These estimates are useful in understanding the magnitude of the problem, but they are broad estimates that are likely to underestimate the total costs associated with acute pain. First, these costs, which were based on 2010 data, are likely an underestimate of total attributable pain costs in more recent years. This underestimation is driven in part by changes in pain management practices following the CDC guidelines, including reduced use of effective pharmacologic treatments for some patients and greater reliance on nonpharmacologic approaches, which may not adequately address severe or moderate acute pain (27). Moreover, there has been a steep decline in the rate of prescription opioid utilization since 2010, and as discussed above it has been shown that the rapid decline in opioid utilization has resulted in unmet pain management needs.

More importantly, these cost estimates omit the costs of opioid-related health problems, including the long-term impact of opioid dependence, misuse, opioid use disorder (OUD), and overdose. For example, one study estimated that the total economic burden of OUD and opioid-related fatal overdoses, which are only a subset of the problems caused by opioids, was approximately $1 trillion, including $92 billion from lost productivity and $23 billion from criminal justice-related expenditures in the U.S. in 2017 (28). In addition, these estimates do not generally account for the costs of undertreatment. Accordingly, this research seeks to address these gaps, which result in a substantial underestimation of the total economic burden of pain. The economic burden of acute pain suggests that there is a need for novel pain treatments that address these gaps, while providing effective pain management with a more balanced safety profile.

## 4 Methods

An analysis was conducted to estimate the annual U.S. economic burden attributable to SAEs related to prescription opioid use and undertreatment of acute pain, including subsequent transition to chronic pain (chronification). In this analysis, costs include direct healthcare costs and indirect productivity losses from a societal perspective. All dollar values reported in published studies were converted to 2024-equivalent U.S. dollars using the Consumer Price Index (CPI). Table 1 summarizes definitions of key terms used throughout the analysis.

**Table 1:** Definition of key terms and sources.

Term	Definition
Serious adverse events	
Misuse/abuse^a^^,^^b^	Any use not directed by a physician, including taking someone else’s prescription, using higher doses or for longer periods than prescribed, or using in any other way not directed by a doctor.
Dependency^a^	In this analysis, dependency is defined as individuals with mild prescription opioid use disorder (POUD). People were classified in the mild POUD category if they met two or three POUD-related DSM-5 criteria in the past 12-months.
Prescription Opioid Use Disorder (POUD)^a^	Includes individuals with moderate and severe POUD. Moderate POUD is defined as someone who met four or five POUD-related DSM-5 criteria in the past 12-months and severe POUD was defined as meeting six or more.
Opioid Use Disorder (OUD)^a^^,^^c^	Individuals who met DSM-5 criteria for opioid use disorder in the past 12 months (excluding POUD)
Nonfatal overdose^d^	A suspected unintentional or undetermined opioid-involved overdose identified through ED visits using syndromic surveillance, based on opioid poisoning diagnosis codes or overdose-related chief complaint text, that did not result in death
Fatal overdoses^e^	A death in which the underlying cause is classified as unintentional, suicide, homicide, or undetermined drug poisoning with opioid involvement identified by multiple cause of death codes. Determinations are based on death certificate data and ICD-10 coding rules.
Undertreatment	
Acute pain^f^	An individual who reports pain occurring on “some days” or “most days” and falls within pain categories 2–4 (moderate to severe).
Chronification^g^	An individual with no pre-existing chronic pain whose pain persists beyond 3 months and occurs on more than 50% of days

a Definition is sourced from the 2023 National Survey on Drug Use and Health (NSDUH) Methodological Summary and Definitions, published by Substance Abuse and Mental Health Services Administration (SAMHSA) NSDUH estimates are weighted to represent approximately 323 million U.S. civilian, noninstitutionalized individuals aged 12 years or older, excluding unsheltered homeless individuals, residents of institutions (e.g., prisons, hospitals, nursing homes), and active-duty military personnel. The final sample consisted of 67,679 completed interviews in 2023.

b Does not include individuals who are reported as having prescription opioid disorder (POUD) or opioid use disorder (OUD).

c Does not include individuals with POUD.

d Definition sourced from Centers for Disease Control and Prevention (CDC) “All Opioid Overdose v4 Parsed” (2024).

e Definition sourced from Centers for Disease Control and Prevention (CDC), National Center for Health Statistics, “Cause of Death and ICD-10 Coding Guidance” (2025).

f Based on Nahin et al. (51). Pain severity was classified using the Washington Group pain categories, a validated, impact-based system combining pain persistence (“some days,” “most days,” or “every day”) and pain “bothersomeness” (“a little,” “between a little and a lot,” or “a lot”). Categories 2–4 were used as a proxy for moderate to severe undertreated acute pain. These categories do not correspond to a numeric pain rating scale (e.g., 0–10).

g Based on Friedman et al. (12), Stephens et al. (14), and Hofer et al. (13). Used as a proxy to define chronification.

### 4.1 Outcomes and populations

Population definitions are consistent with those established by SAMHSA and NSDUH criteria. Categories included in this analysis are misuse/abuse, dependency, prescription-opioid use disorder (POUD), opioid use disorder (OUD), and fatal and nonfatal overdoses. Misuse and abuse refer to any use not directed by a clinician (e.g., non-prescribed use, higher dose/frequency than prescribed, or non-medical use). In this analysis, dependency is defined as mild POUD. Under DSM-5 criteria, POUD severity is based on the number of criteria met in the past 12 months (mild: 2–3 criteria; moderate: 4–5 criteria; severe: ≥6 criteria). NSDUH 2023 defines POUD as meeting 4 or more DSM-5 criteria for prescription pain-reliever misuse; therefore, only moderate and severe POUD are included in the POUD category here. OUD follows NSDUH DSM-5 criteria for heroin or prescription opioid use disorder, with POUD cases excluded to prevent overlap. Fatal overdose represents opioid-involved mortality, and nonfatal overdoses are opioid related overdoses that did not result in death.

Undertreatment categories include overall acute pain that is under-treated, defined as persistent moderate-to-severe pain occurring on some or most days (not every day). Chronification is defined as new persistent pain lasting >3 months among individuals without pre-existing chronic pain. To avoid double counting, chronification cases are subtracted from the undertreated acute-pain pool.

### 4.2 Prevalence

The 2023 National Survey on Drug Use and Health (NSDUH) reported by the Substance Abuse and Mental Health and Services Administration (SAMHSA) was used to estimate prevalence of SAE categories, with an exception for nonfatal overdose estimates which were derived from CDC estimates (29). No distinct prevalence estimates for dependency are provided in the literature, therefore, dependency was defined using counts of mild POUD, while the POUD category was restricted to moderate and severe POUD as reported by NSDUH. To ensure mutually exclusive categories and avoid double counting, misuse excludes all POUD and OUD cases, and the OUD category excludes POUD cases.

Nonfatal overdose prevalence was estimated using the CDC-reported rate of 15.6 nonfatal opioid overdoses per 10,000 all-cause emergency department (ED) visits in 2024, calculated across 46 participating jurisdictions using data from the Drug Overdose Surveillance and Epidemiology Discharge Surveillance (DOSE-DIS) system (29). This rate was applied to total U.S. ED visit volume to estimate national ED nonfatal overdose counts. To allocate nonfatal overdose events between ED and inpatient settings, the distribution of overdose-related discharges reported to DOSE-DIS (131,620 ED visits and 54,044 inpatient hospitalizations) was used to derive the proportion of events occurring in each care setting. These proportions were applied to the national ED overdose estimate to generate corresponding inpatient counts. All estimates were scaled to national values by applying CDC-reported rates to the total number of U.S. emergency department visits, accounting for partial state participation in DOSE-DIS.

Prevalence of undertreated acute pain was derived by first restricting national pain survey estimates to cases of moderate and severe acute pain and then applying literature-based estimates of undertreatment that exclude chronification. Rates of undertreatment have been reported to be about 35% (12, 14, 30). Chronification prevalence was calculated by applying an estimated 22% transition rate from undertreated acute pain to chronic pain, based on literature estimates (12–14). Individuals estimated to transition to chronic pain were explicitly removed from the undertreated acute pain category, ensuring that acute undertreatment and chronic pain outcomes were treated as mutually exclusive to prevent double counting.

### 4.3 Costs

#### 4.3.1 Healthcare costs

Direct medical costs per case were obtained from peer-reviewed studies across opioid SAE and undertreatment categories. The estimates of incremental healthcare costs for misuse and dependent cases reported in these studies were derived from previous analyses of claims data, rather than new analyses conducted for this study. All reported cost estimates were inflated to 2024 USD. These costs account for prescription, outpatient, and inpatient-related costs (52). Inputs for OUD and POUD are estimated using data from the CDC’s analysis of state-level OUD-related healthcare costs, as well as costs associated with fatal overdoses (31). Nonfatal overdose costs combined EMS costs, ED discharge costs, and inpatient hospitalization costs for nonfatal overdose events in addition to expected costs of overdose-related morbidity sequelae (32). Morbidity costs were weighted by the probability of each outcome and applied separately for ED-treated and inpatient-treated cases (e.g., chest infection, pneumonia, nerve palsy, brain injury). Undertreated acute pain costs were estimated using a proxy measure based on annual costs associated with newly diagnosed low back and lower extremity pain, including imaging, procedures, and professional visits (33). For the chronification category, per-case chronic pain costs were derived by applying published estimates of total U.S. healthcare costs attributable to chronic pain to the estimated prevalence of chronic pain, based on study by Gaskin and Richard. (2).

#### 4.3.2 Productivity costs

The analysis of indirect costs associated with SAEs was derived primarily from literature focused on OUD and fatal overdose-related productivity costs (31). For misuse, we assumed that costs were 50% of the OUD costs. This assumption is consistent with prior estimates of the societal costs of prescription opioid misuse and dependence (34). For undertreated acute pain categories, productivity losses were estimated using absenteeism and presenteeism estimates from pain-condition studies (2, 53).

## 5 Results

Table 2 summarizes the annual cost burden across opioid-related serious adverse events and undertreatment of acute pain, totaling approximately $425 billion in 2024 US dollars. The equations and general calculation approach used to derive these estimates are provided in the Technical Appendix. When disaggregated by cost category, indirect costs associated with productivity accounted for approximately 74% of total economic burden ($314B), while direct healthcare expenditures comprised the remaining 26% ($110.4B). Grouped by health outcome category, serious adverse events represented approximately 83% of total costs, with undertreatment of acute pain accounting for the remaining 17%. Within the serious adverse event category, fatal overdoses were the largest contributor ($150.4B; 35.4% of total cost), followed by dependency ($64.4B; 15.2% of total cost), and POUD ($57.7B; 13.6% of total cost). Of the costs attributable to undertreatment, $27.0B (6.4% of total costs) were associated with undertreated acute pain and $46.6B (10.9%) with chronification of pain. Although nonfatal overdoses were more frequently treated in the ED compared to the inpatient setting, inpatient cases accounted for approximately 36% of nonfatal overdose costs due to substantially higher average costs.

**Table 2:** Summary of estimated costs attributable to serious adverse events and undertreatment, 2024.

Outcome	Prevalence (*n*, 000 s)	Excess healthcare avg. cost per case ($)	Excess productivity avg. cost per case ($)	Total excess costs (millions $)
Serious adverse events				
Misuse/abuse	2,942 (600–11,800)	$8,910 ($6,000–$15,000)	$9,414 ($5,000–$15,000)	$53,909 ($6,600–$354,000)
Dependency	3,534 (3,000–5,300)	$8,815 ($6,000–$14,000)	$9,414 ($6,000–$20,000)	$64,415 ($36,000–$180,200)
POUD	1,450 (1,160–1,740)	$20,952 ($15,000–$30,000)	$18,828 ($12,000–$40,000)	$57,695 ($31,320–$121,800)
OUD	400 (360–500)	$20,952 ($15,000–$30,000)	$18,828 ($12,000–$40,000)	$15,912 ($9,720–$35,000)
Nonfatal overdose (ED)	242 (180–580)	$3,416 ($2,000–$5,000)	$20,407 ($10,000–$40,000)	$5,776 ($2,160–$26,100)
Nonfatal overdose (Inpatient)	85 (60–130)	$18,073 ($12,000–$30,000)	$20,467 ($12,000–$40,000)	$3,272 ($1,440–$9,100)
Fatal overdoses	81 (42–100)	$7,035 ($5,000–$15,000)	$1.85 million ($1.2–$3 million)	$150,386 ($50,610–$301,500)
Undertreatment				
Acute pain	3,633 (2,500–4,700)	$1,348 ($800–$2,500)	$6,090 ($3,000–$12,000)	$27,024 ($9,500–$68,150)
Chronification	1,005 (700–1,300)	$6,369 ($4,500–$12,000)	$40,035 ($20,000–$80,000)	$46,633 ($17,150–$119,600)
Total				$425,021 ($164,500–$1,215,450)

Values in parentheses denote the lower and upper bounds of estimates reported across the literature. POUD = prescription opioid use disorder; OUD = opioid use disorder; ED = emergency department.

To account for variation around these estimates, Table 2 also reports lower- and upper-bound ranges that are informed by published prevalence, cost, and utilization estimates from the literature. Ranges were informed by representative surveys, systematic reviews, and cost-of-illness studies. The available literature does not consistently provide nationally representative, U.S. population-level estimates across all opioid-related outcomes and undertreatment categories. Instead, much of the evidence base focuses on specific subpopulations such as adolescents, older adults, surgical cohorts, veterans, Medicaid enrollees, or individuals with prior overdose, which contributes to substantial variation in reported prevalence across studies (35–37). As a result, observed prevalence estimates vary significantly depending on the population under study, with higher values often observed in younger or higher-risk groups and lower values in older or general adult samples.

Cost estimates similarly vary across the literature, reflecting differences in care setting, severity, payer mix, and perspective. Excess healthcare costs differ substantially between ED-treated and inpatient-treated cases, while productivity losses vary widely depending on assumptions about employment, disability, and premature mortality (38–40). To further assess how uncertainty in key parameters affects these estimates, a one-way sensitivity analysis was conducted to evaluate the impact of variation in individual inputs on total cost and identify the primary cost drivers. The analysis showed that total cost estimates are most sensitive to variation in misuse and abuse prevalence, followed by overdose-related productivity costs and prevalence. Results are presented in the Technical Appendix (Figure B1) as a tornado diagram, which displays the range of total cost estimates associated with each parameter relative to the base-case value. Across all parameter variations in the one-way sensitivity analysis, total cost estimates ranged from approximately $352 billion to $587 billion (−17 to +38% relative to the base-case estimate of $425 billion), indicating that overall estimates remain relatively stable despite variation in individual inputs.

## 6 Discussion

### 6.1 Direct healthcare costs and lost productivity

The estimated annual economic burden of $425.0 billion ($164.5–$1,215.45B, in 2024USD) observed in this analysis reflects the substantial clinical and economic complexity associated with opioid-related serious adverse events and undertreatment of acute pain. Much of the direct healthcare burden was associated with dependency, misuse/abuse, opioid use disorders (OUD), and chronification of acute pain. Among individuals with OUD/POUD, excess healthcare costs may in part reflect the more frequent healthcare interactions associated with high relapse rates, co-occurring mental health conditions, and increased risk of serious bacterial infections that often require long-term intravenous antibiotics and prolonged hospitalization (20, 41). Consequently, individuals at risk of dependence, misuse, and progression to OUD or POUD are expected to have higher utilization of inpatient care, emergency department services, mental health services, and intensive behavioral health treatment. Similarly, failure to achieve adequate pain resolution has profound economic and clinical consequences. Indeed, persistent postoperative pain is a strong predictor of costly readmissions and increased emergency department visits, highlighting a critical need for therapeutics that can interrupt the progression toward chronification and its associated surge in healthcare resource utilization (42). In contrast, lost productivity costs are dominated almost entirely by fatal overdoses, primarily because of premature mortality. The concentration of productivity losses within fatal overdose and chronification categories suggests that a substantial portion of the economic burden associated with pain management may arise outside traditional healthcare expenditures and extend into longer-term societal and workforce impacts, particularly in the context of chronification-related absenteeism, presenteeism, reduced labor force participation, and long-term disability.

### 6.2 Societal costs associated with OUD and pain chronification

Other indirect costs not accounted for in this analysis include crime related costs. There is growing evidence of association between opioid exposure and higher rates of crime. For example, states more heavily exposed to introduction of OxyContin experienced a 25% increase in violent crime and 12% increase in property crime, relative to states with more restrictive prescription monitoring (43). Opioid use disorders have also been associated with higher rates of criminal justice involvement over time (44, 45). Prior estimates that include crime-related costs, place the total economic burden of OUD and opioid-related fatal overdoses at nearly $1 trillion, meaning the total burden presented here is likely an underestimate.

Beyond the economic data, the humanistic toll of pain represents a profound crisis for the individual and their families. For the individual, reliance on opioids to treat severe pain introduces the risk of iatrogenic harm, including the development of serious adverse events and potential dependency. At the family level, the economic toll extends beyond simple medical expenditures. Family caregivers of individuals with OUD face significant subjective and objective burdens including emotional distress, financial instability, and social stigma that threaten their physical and mental well-being (46).

On the other end, the failure to adequately manage severe acute pain significantly elevates the risk of chronification, a transition that not only diminishes quality of life (QoL) and drives disability but also precipitates psychological wellbeing. Indeed, over 55% of adults with chronic pain suffer from unremitted comorbid anxiety and depression, a prevalence nearly six times higher than those without chronic pain, often resulting in loss of personal identity, social withdrawal, and an increased inability to perform basic daily activities (47). The chronification of pain also creates a cascade effect on the family, where individual functional limitations eventually destabilize the entire family system. This high family impact affects approximately 11 million U.S. adults and is characterized by a six-fold increase in comorbid mental health symptoms and the disruption of essential household roles, often forcing children into caregiving capacities (47). Collectively, these struggles represent a massive societal drain and further highlight the critical need for high-efficacy interventions that preserve patient function while mitigating long-term disability.

These findings highlight the limitations of approaches focused primarily on reducing opioid exposure without adequately addressing effective pain management leaving the United States with both a prescribing problem as well as an over-correction problem. Although utilizing opioids for moderate-to-severe pain increases the risk of costly adverse events, the significant burden attributable to chronification observed in this analysis indicates that undertreatment of acute pain may itself represent an important contributor to long-term healthcare utilization and productivity loss. Opioid risks cannot be eliminated and are inherent to their use, and effective alternatives for many acute pain episodes do not yet exist. Thus, policy changes or improved treatment algorithms can only improve this situation on the margins. Addressing this burden will require the development of novel therapies that can treat pain as well as opioids with a lower risk of misuse, dependence, addiction, and overdose.

## 7 Limitations

There are several limitations to this analysis. First, although we contend that it is important to differentiate between OUD stemming from prescription opioids versus illicit opioids, the literature differentiating POUD and OUD is somewhat limited, especially with regard to costs. Consequently, estimates of costs associated with POUD versus OUD differ only in the underlying epidemiology rather than the costs per case. The study also relies on proxy estimates for undertreated acute pain (e.g., newly diagnosed low back and lower extremity pain), which may limit generalizability to other pain conditions. Several cost inputs are derived from dated or incomplete surveillance data. Given the profound changes in the volume and mix of prescription and illicit opioid use over the past decade, the estimates of specific components of costs used in this study could overstate or understate estimates that might be obtained by replicating the dated cost studies with current data. Some nonfatal overdose cases are likely not to be captured, such as those treated by EMS but not resulting in ED visits or hospitalizations. Despite efforts to minimize double counting across SAE categories, residual overlap remains a possibility. Finally, the absence of longitudinal follow-up of POUD cases and prescription opioid users in existing datasets limits the ability to fully capture the long-term progression between prescribing patterns, adverse events, and chronic pain outcomes.

## 8 Conclusion

Our findings identify overdoses and chronification cases as significant drivers of the economic burden of pain, indicating two sides of a single systemic problem. Addressing this burden in full is more critical than ever and will require innovative treatments that provide effective pain relief without risk of opioid-related consequences.

## Footnotes

1 Data reported by authors in 2010 dollars, trended forward to 2024 using the U.S. Consumer Price Index (“CPI”).
