# Supplementary material for: The Economic burden of opioid-related serious adverse events and undertreatment of acute pain in the United States
Source: Front Public Health. 2026 Jun 17;14:1824038. doi: 10.3389/fpubh.2026.1824038 (PMC13319003; doi:10.3389/fpubh.2026.1824038)
Supplement: Supplementary file 1 [file Supplementary_file_1.pdf]

Technical Appendix

This appendix provides additional detail on cost calculations and the one-way sensitivity analysis used to assess the impact of parameter uncertainty on total cost estimates.

A. Calculation of Total Cost Estimates

1. Population and Case Estimation
   - Number of cases = population * prevalence (condition)
2. Category Definitions and Adjustments
   - Misuse cases = total misuse – POUD – OUD
   - OUD cases = total OUD – POUD
   - Dependency cases = mild POUD cases
   - POUD cases = moderate + severe POUD cases
   - Undertreated acute pain = total undertreated acute pain – chronification cases
3. Nonfatal Overdose Estimation
   - Total nonfatal overdoses = overdose rate per ED visit * total ED visits
   - ED-treated Overdoses = total nonfatal overdoses * proportion treated in ED
   - Inpatient-treated overdoses = total nonfatal overdoses * proportion treated inpatient
   - National estimates = reported rates * total U.S. ED visits
4. Undertreatment and Chronification
   - Undertreated acute pain cases (initial) = population * prevalence of moderate-to-severe acute pain * undertreatment rate
   - Chronification cases = undertreated acute pain cases * transition rate to chronic pain
   - Undertreated acute pain cases (final) = undertreated acute pain cases – chronification cases
5. Direct Healthcare Costs
   - Healthcare cost (condition) = number of cases * excess healthcare cost per case
   - Excess healthcare cost per case = cost with condition – baseline cost
6. Nonfatal Overdose Cost Components
   - Total nonfatal overdose cost per case = EMS cost + ED cost + inpatient cost + morbidity cost
   - Morbidity cost = sum of (probability of complication * cost of complication)
7. Chronification Cost Estimation
   - Cost per chronic pain case = total national chronic pain cost / total chronic pain prevalence
   - Chronification cost = chronification cases * cost per chronic pain case
8. Inflation Adjustment
   - Cost (2024 USD) = reported cost * CPI adjustment factor
9. Productivity Costs
   - Productivity cost (condition) = number of cases * productivity cost per case
   - Productivity cost (misuse per case) = 50% * productivity cost (OUD per case)
10. Total Cost Estimation
    - Total cost (condition) = healthcare cost + productivity cost
    - Total economic burden = sum of total costs across all conditions

B. One-Way Sensitivity Analysis

A one-way sensitivity analysis was conducted by varying each parameter individually across its lower- and upper-bound values while holding all other inputs constant at their base-case values to assess the impact of parameter uncertainty on total cost estimates. Results are presented in Figure B1 as a tornado diagram, which ranks parameters based on the magnitude of their impact on total cost. The diagram displays the range of total cost estimates associated with each parameter relative to the base-case value. The analysis shows that total cost estimates are most sensitive to variation in misuse and abuse prevalence, followed by fatal overdose-related productivity costs and prevalence, which produce the largest variation in overall economic burden. In contrast, parameters related to nonfatal overdose events and certain healthcare cost inputs have relatively smaller effects on total cost.

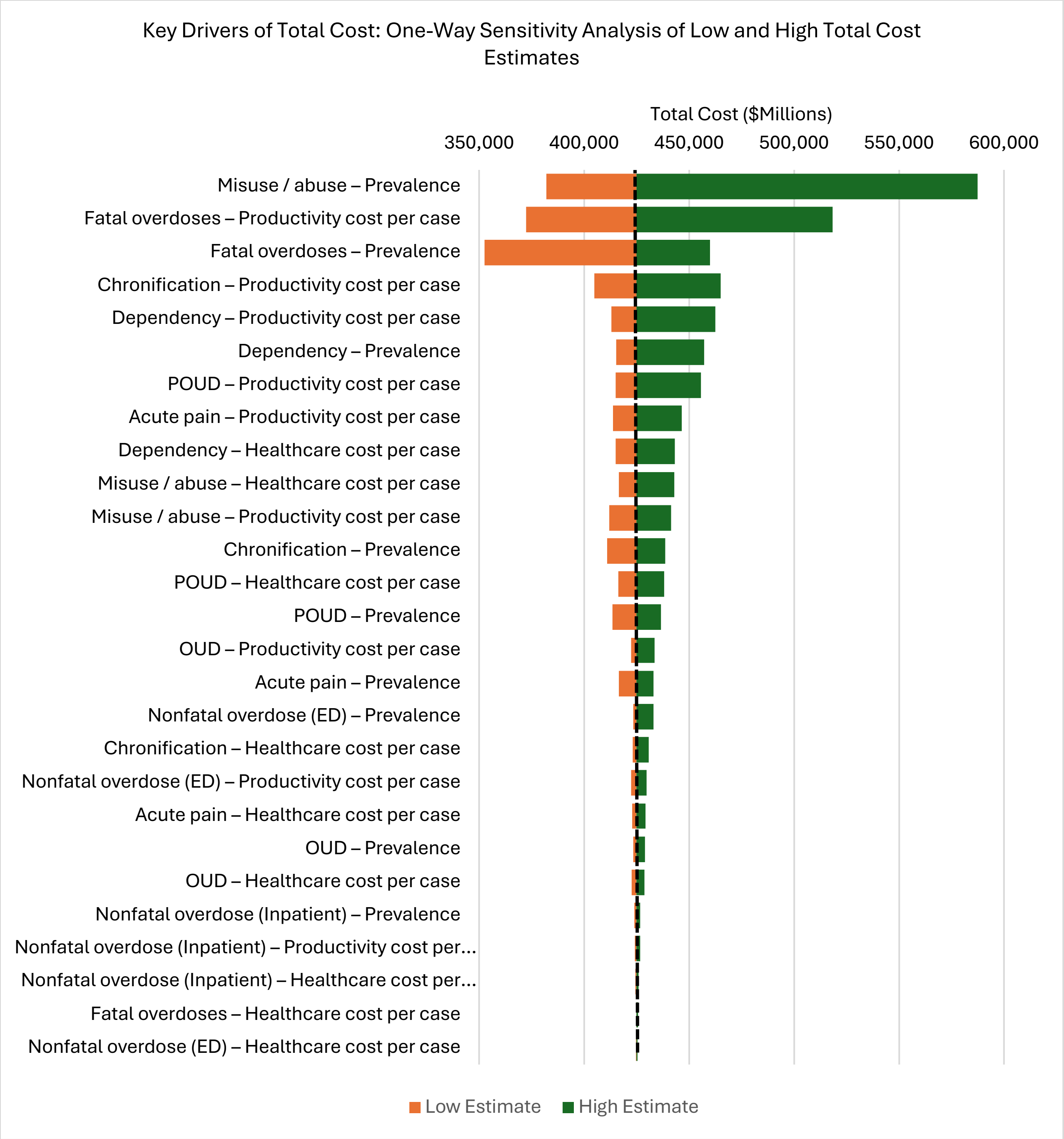

Figure B1. Tornado Diagram of One-Way Sensitivity Analysis of Total Cost
